# Distinct and stage specific nuclear factors regulate the expression of falcipains, *Plasmodium falciparum *cysteine proteases

**DOI:** 10.1186/1471-2199-9-47

**Published:** 2008-05-14

**Authors:** Sujatha Sunil, Virander S Chauhan, Pawan Malhotra

**Affiliations:** 1 Malaria Group, International Centre for Genetic Engineering and Biotechnology, PO Box 10504, Aruna Asaf Ali Marg, New Delhi 110067, India

## Abstract

**Background:**

*Plasmodium falciparum *cysteine proteases (falcipains) play indispensable roles in parasite infection and development, especially in the process of host erythrocyte rupture/invasion and hemoglobin degradation. No detailed molecular analysis of transcriptional regulation of parasite proteases especially cysteine proteases has yet been reported. In this study, using a combination of transient transfection assays and electrophoretic mobility shift assays (EMSA), we demonstrate the presence of stage specific nuclear factors that bind to unique sequence elements in the 5'upstream regions of the *falcipains *and probably modulate the expression of cysteine proteases.

**Results:**

Falcipains differ in their timing of expression and exhibit ability to compensate each other's functions at asexual blood stages of the parasite. Present study was undertaken to study the transcriptional regulation of *falcipains*. Transient transfection assay employing firefly luciferase as a reporter revealed that a ~1 kb sequence upstream of translational start site is sufficient for the functional transcriptional activity of *falcipain*-1 gene, while *falcipain*-2, -2' and -3 genes that exist within 12 kb stretch on chromosome 11 require ~2 kb upstream sequences for the expression of reporter luciferase activity. EMSA analysis elucidated binding of distinct nuclear factors to specific sequences within the 5'upstream regions of *falcipain *genes. Analysis of *falcipains' *5'upstream regulatory regions did not reveal the presence of sequences known to bind general eukaryotic factors. However, we did find parasite specific sequence elements such as poly(dA) poly(dT) tracts, CCAAT boxes and a single 7 bp-G rich sequence, (A/G)NGGGG(C/A) in the 5' upstream regulatory regions of these genes, thereby suggesting the role(s) of *Plasmodium *specific transcriptional factors in the regulation of *falcipain *genes.

**Conclusion:**

Taken together, these results suggest that expression of *Plasmodium *cysteine proteases is regulated at the transcriptional level and parasite specific factors regulate the expression of *falcipain *genes. These findings open new venues for further studies in identification of parasite specific transcription factors.

## Background

*Plasmodium falciparum*, a human malaria parasite has a complex life cycle that encompasses three major developmental stages; the mosquito, liver and blood stages. During the complex cycle of *P. falciparum*, the intracellular development of the different asexual and sexual stages proceeds through a dynamic and multistep process for which the parasite has evolved complex molecular strategies [1]. *P. falciparum *proteome data show a considerable level of regulation of gene expression throughout its life cycle; only 6% of the proteins identified are expressed at all the four stages. The transcriptome analysis of *Plasmodium falciparum *has revealed that the parasite has evolved an extremely specialized mode of transcriptional regulation at asexual blood stages that produces a continuous cascade of gene expression, beginning with genes corresponding to general cellular processes and ending with genes with specialized functions, such as genes involved in erythrocyte invasion [2]. The transcriptome of intraerythrocytic developmental cycle (IDC) of *PIasmodium falciparum *thus resembles a "just-in-time" manufacturing process whereby transcripts are essentially produced when required. This concept has also been referred as "transcripts to go model" [3]. For example, merozoite surface proteins that are required for erythrocyte invasion are expressed mainly at late schizont and merozoite stages [2,4].

Transcriptional regulation and post-transcriptional gene silencing through translational repression of messenger RNA have been shown to be the major regulatory mechanisms in *P. falciparum *[4-6]. Malaria parasites express structurally distinct sets of rRNA genes in a stage specific manner that supposedly alter the properties of the ribosomes and thus modify patterns of cell growth and development [7]. Transcriptional regulation is the major regulatory mechanism that determines the expression of one *var *gene and silencing of other *var *genes in a single parasite. [8]. Regulatory regions within the *var *gene promoters have been shown to determine their silencing; their introns act as transcriptional silencing elements that help to control antigenic variations [9,10]. Post-transcriptional regulation has been shown for P28, a gametocyte specific protein that remains in a state of translational repression in developing and mature gametocytes [11]. Based on these studies, it has been proposed that the developmental stages of malaria parasite require coordinated modulation of expression of distinct sets of genes, which could be achieved by transcriptional and/or post-transcriptional control. In *Plasmodium *species, as in all eukaryotes, gene expression is governed at the level of transcription by the interaction of elements within promoters acting in *cis *(DNA regulatory elements) and/or *trans *whose availability is modulated during *P. falciparum *development [12]. However, little is currently known about these elements in *Plasmodium *species. Insights into the regulatory pathways of malaria parasites will lead to better understanding of *Plasmodium *biology and development of new chemotherapeutics and vaccine strategies.

*P. falciparum *expresses four cysteine proteases namely, falcipain-1 (FP-1), falcipain-2 and -2' (FP-2A and FP-2B) and falcipain-3 (FP-3) at asexual blood stages of the parasite. These proteases perform multiple functions such as hemoglobin hydrolysis, erythrocyte rupture and erythrocyte invasion and differ in their timing of expression. Falcipain-1 is active at the invasive merozoite stage while falcipain-2/-2' and -3 are expressed mainly at early and late trophozoite stages respectively [13-15]. A recent falcipain-2 knockout study has suggested interplay between different cysteine proteases [16]. In the present study, we evaluated the mechanism of gene regulation of *falcipains*. We identified specific promoters regions of the four *falcipains *and observed that distinct nuclear factors bind to these *falcipains *promoters in a stage specific manner. We also observed stage specific expression of these nuclear factors, thereby suggesting a gene specific transcriptional regulation of *falcipains*.

## Results

### Identification of transcription start sites for the falcipain genes

**Figure 1:**
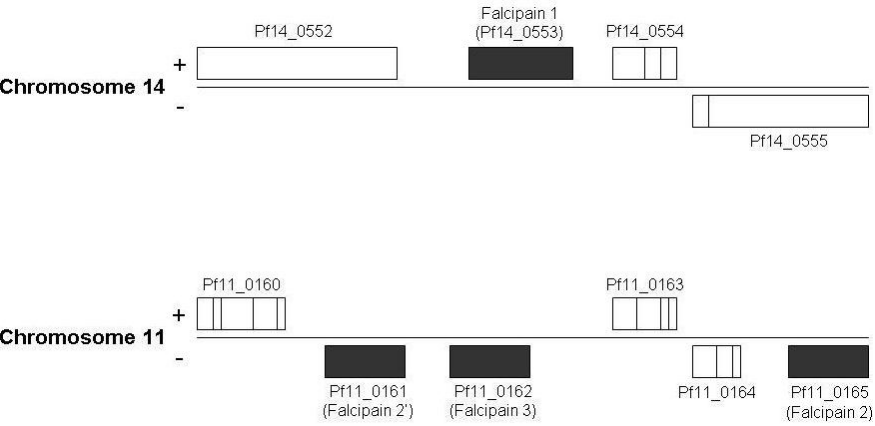
**Genomic organization of *P. falciparum *genes**. A schematic representation of *Falcipain*-1, -2, -2' and -3 genes with the neighboring genes and their PlasmoDB IDs. *Falcipain *genes are indicated as grey boxes. The orientation of the genes are denoted by +/- signs.

**Figure 2:**
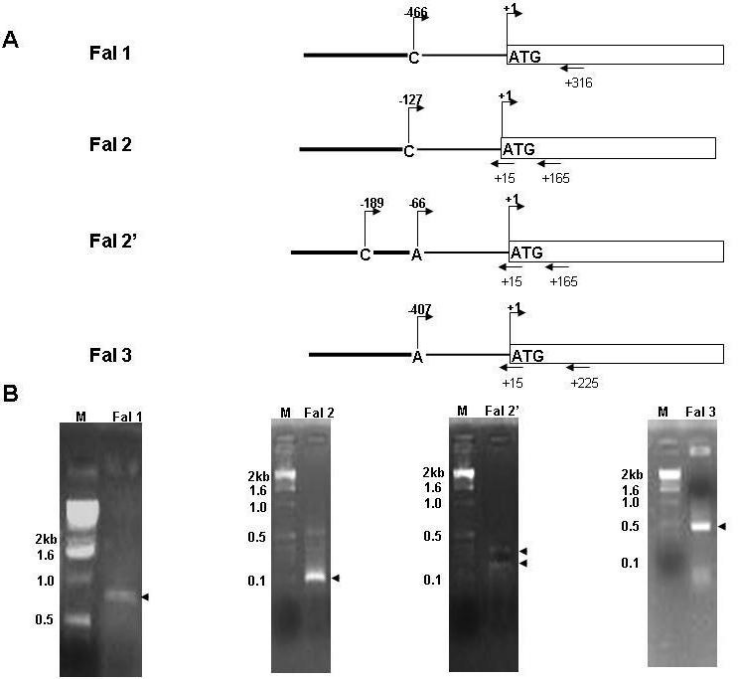
**Mapping of *falcipains *transcription start site**. (A) Schematic representation of the non-coding region of the *falcipains *showing the transcription  and translation start sites. Positions downstream of the ATG are denoted with a (+) sign and those upstream with a (-) sign. Thick lines denote regions before the transcription start site. Thin lines denote regions after the start site. Arrows denote the primers and their positions used for 5'RACE. (B) Agaorse gel electrophoresis of 5'RACE products. Lane M, DNA size markers. Arrows denotes the PCR amplified products.

Four *falcipain *genes are encoded on two different chromosomes; *falcipain-1 *is encoded on chromosme 14, while three other *falcipains *(*falcipain-2, -2' and -3*) are located within a 12 kb stretch of chromosome 11 (Fig. 1). Among the three *falcipains *on chromosome 11, *falcipain-2' *and *3 *exist in tandem, an intragenic region of 1.6 kb separates them, while *falcipain-*2 lies further upstream. In an attempt to understand the regulation of these cysteine proteases, we carefully analyzed the 5'upstream regions of *falcipain *genes. Firstly, we identified the transcriptional start site of all the *falcipains *(Fig. 2A). The location of the transcriptional start site for each of the *falcipains *was determined by 5'RACE analysis using gene specific primers as described in Table 1. Falcipain 2 and 2' share a 97% homology and are different only at three amino acids positions. The 5' upstream regions however, are quite varied. The gene specific primers (GSP1 and GSP2) were the same for these falcipains, while GSP3 was designed immediately upstream to the translational start site and was essential for determining the transcriptional start sites of *falcipain 2 *and 2'. Following RACE, a single PCR product was observed for *falcipain*-1,-2 and -3 genes, while two fragments were seen in case of *falcipain*-2' gene indicating the existence of two start sites in this gene (Fig. 2B). The amplified products were cloned into a TA cloning vector and sequence analysis was carried out to determine the exact position of the transcription initiation sites (+1) for the four *falcipains*. Transcription start sites for *falcipain*-1, -2, -2' and -3 were at nucleotide positions -466, -127, -189 and -407 upstream of the translational start site. Figure 2A depicts the exact position of start sites for different *falcipain *genes.

**Table 1:** List of Primers and PCR conditions used for 5' RACE

**Gene**	**Primers**	**Primer sequences 5'-3'**	**PCR conditions**		
			
			**Denaturation**	**Annealing**	**Extension**
**Falcipain 1**	Fal1RACE GSP1R	CAAGATAGGATAGAAGACTTCCTC			
	Fal1RACE GSP2R	CATCTTCTTCATTGGAGGATTC	94°C 1'	52°C 1.30'	68°C 1.30'
	Fal1RACE GSP3R	CCATGTTGTAATCCATGGTCTTCAAC	94°C 1'	50°C 1.30'	68°C 1.30'
**Falcipain 2**	Fal2RACE GSP1R	TTACCTTAATGGAATGAATGCATC			
	Fal2RACE GSP2R	TAGGAAAATAAAATAAAACAAAACCAA	94°C 1'	52°C 1'	68°C 1.30'
	Fal2RACE GSP3R	CATGTGGTAATCCATGGTTAAAA	94°C 1'	52°C 1.30'	68°C 1.30'
**Falcipain 2a**	Fal2aRACE GSP1R	TTACCTTAATGGAATGAATGCATC			
	Fal2aRACE GSP2R	TAGGAAAATAAAATAAAACAAAACCAA	94°C 1'	52°C 1'	68°C 1.30'
	Fal2aRACE GSP3R	TATGAATATACATATACACTAGG	94°C 1'	50°C 1.30'	68°C 1.30'
**Falcipain 3**	Fal3RACE GSP1R	TTCAAGTAATGGTACATAAGCTTC			
	Fal3RACE GSP2R	CCTAGGATCGTTTATATTATTTGATAA	94°C 1'	52°C 1'	68°C 1.30'
	Fal3RACE GSP3R	CATATGATATTCCATGGTTCAAAC	94°C 1'	50°C 1.30'	68°C 1.30'

### Identification of promoter regions of falcipain genes

**Figure 3:**
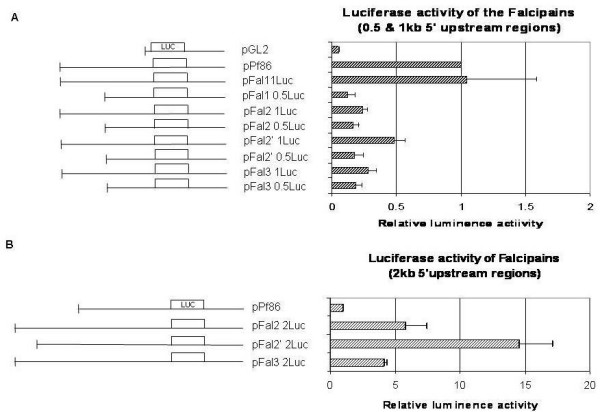
**Functional analysis of *falcipain *promoter constructs. The plasmids are shown in the left. Numbers indicate the size of 5' sequence of respective *falcipain *used in each plasmid**. (A) Ring stage parasites were transfected with 75 μg of plasmids containing the 0.5 and 1 kb constructs of the *falcipains*. Co-transfection with 75 μg of *renilla *served as internal control for the transfection assays. Vector pGL2 (Promega) served as negative control. The luciferase gene is indicated as box. Percentage relative luciferase activity was calculated by normalizing the luciferase *firefly *to the *renilla *internal control values. The values shown represent the mean ± standard deviation of two independent transfection experiments performed in triplicates. (B) Ring stage parasites were transfected with 75 μg of plasmids containing 2 kb constructs of the *falcipains*.

To define the functional promoter sequences required for *falcipain *gene expression, 0.5 kb and 1 kb 5'-upstream regions of all the four *falcipains *were PCR amplified. These intergenic fragments were cloned upstream of the *firefly *luciferase gene in pPf86, a plasmid designed for reporter gene expression in *P. falciparum*. These plasmids (pPfal1 .5Luc, pFal1 1Luc, pFal2 1Luc, pFal2' 1Luc and pFal3 1Luc) were co-transfected with plasmid pPfrluc (in which the 5'CAM promoter is fused to the *renilla *luciferase ORF). Pfrluc served as an internal control to nullify the variations in transfection efficiencies. Vector pGL2 (Promega) served as a negative control. This vector had the luciferase gene with no promoter sequence upstream of it. Transfections of the plasmids were carried out at ring stage. -1032 bps 5'upstream regulatory region of *falcipain-*1 gene (pFal1.1) was able to drive the reporter gene expression comparable to 5'region of pPf86 vector (positive control), however corresponding regions of *falcipains*-2 (-1042 bps), 2' (-1033 bps) and -3 (-1035 bps) genes produced low levels of reporter activities. Fig. 3A shows the reporter activities after 48 h of transfection (ring stage) of all the constructs. Expression plasmids possessing truncated versions of each 5' upstream sequence (0.5 kb) showed significantly low level of expression (Fig. 3A). These results indicated that ~1 kb 5'upstream region of *falcipain-*1 constituted a functional promoter, while corresponding sequences in other *falcipain *genes exhibited lower level of luciferase expression that may represent basal promoter activity.

To identify the functional promoter sequences for *falcipain-2, -2' and -3 *genes, we next cloned ~2 kb 5'-untranslated regions of these genes in pPf86 plasmid. In *falcipain 2 and 3*, regions spanning -1882 bps, -1903 bps respectively from the translational start site were cloned into pPf86 vector. In case of *falcipain-2' *gene, 1610 bps of intergenic region between *falcipain-*2' and *falcipain-*3 was cloned into the luciferase vector. Each plasmid was co-transfected with pPfrluc into *P. falciparum *ring stage parasites. Fig. 3B shows the reporter gene expression derived from 1.6/2 kb 5' upstream regulatory regions of three *falcipain *genes. A high level of luciferase activity was observed for the three *falcipains *promoters. The 5'upstream regulatory region of *falcipain*-2' gene exhibited 2–3 fold higher luciferase activity than that exhibited by the corresponding sequences of *falcipains-*2 and 3 genes. Together, our data suggests that approximately 2 kb long 5'upstream sequences are required for functional promoter activity of *falcipain*s -2, -2' and -3.

### Analysis of falcipains promoter activities at different asexual blood stages

**Figure 4:**
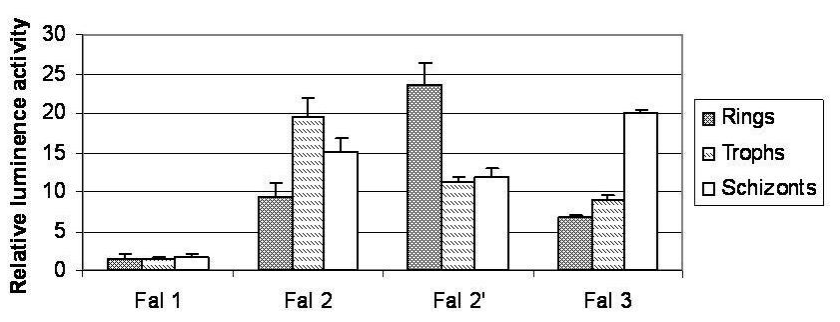
**Luciferase activity of plasmid constructs showing the reporter gene activity at the three asexual blood stages of *P. falciparum*.** Ring stage parasites transfected with 75 μg of plasmids containing the 1 kb construct of *falcipain *1 and 2 kb constructs of *falcipain2, 2' and 3*. Co-transfection with 75 μg of *renilla *served as internal control for the transfection assays. Luciferase assays were performed at 48 h, 75 h, 96 h post transfection. The luciferase gene is indicated as box. Percentage relative luciferase activity was calculated by normalizing the luciferase *firefly *to the *renilla *internal control values. The values shown represent the mean ± standard deviation of two independent transfection experiments performed in triplicates.

Falcipains differ in their timing of expression at the three asexual blood stages of *P. falciparum *and what would be informative is to find out if the promoters were functional in a stage specific manner. For this purpose, we carried out the luciferase activity assay for the *falcipains *5' upstream sequences at the three asexual blood stages. As shown in fig. 4, functional promoter activities for all the four *falcipains *were detected at ring, trophozoite and schizont stages, however marked differences were observed in the expression of reporter gene at the three stages. *Falcipain*-2 (pFal2 2Luc), *falcipain*-3 (pFal3 2luc) and falcipain -2' (pFal2' 2Luc) showed maximum luciferase activities at the trophozoite, schizont and ring stages respectively (Fig. 4). The results of stage specific promoter activities of *falcipain*-2 & 3 genes coincided with the timing of expression of these proteins i.e. falcipain-2 and -3 are maximally expressed at the trophozoite and schizont stages respectively. Promoter activity of *falcipain-1 *between the three asexual blood stages was comparable with a slight upsurge at the schizont stage.

### Specific nuclear factors bind to falcipains 5' upstream regulatory sequences

**Figure 5:**
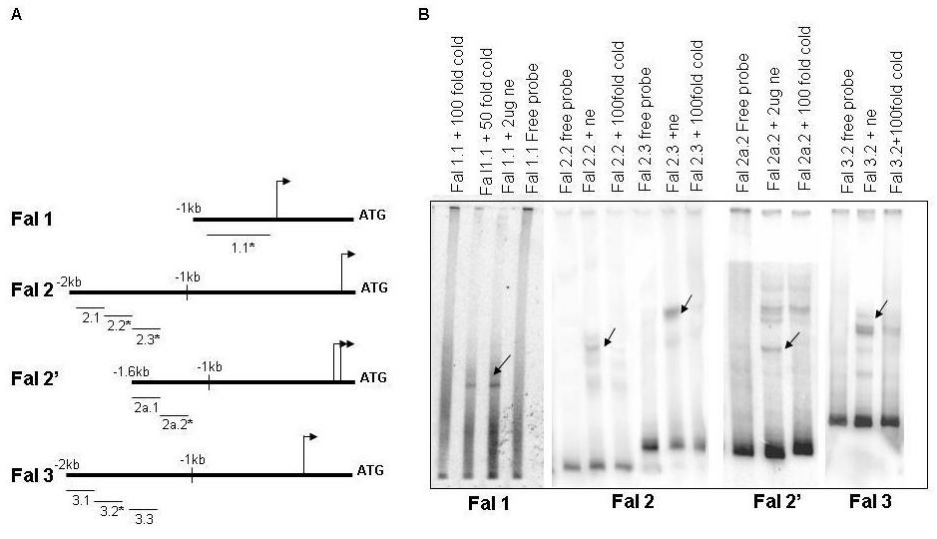
**Sequence specific interaction of 5'upstream regulatory sequences of the *falcipain *genes with parasite nuclear factors**. (A) Schematic representation of the probes used for EMSAs. Short lines indicate the relative positions of double stranded nucleotide probes used in EMSA. Astericks denote those probes that formed complexes with nuclear extracts. (B) Electrophoretic mobility shift assay. End labeled double-stranded oligonucleotides were incubated with 2–5 μg of nuclear proteins isolated from asynchronous parasite culture. For competition experiments, a 100 fold excess of the respective unlabelled double-stranded oligonucleotides were used. Arrows indicate the positions of DNA-protein complexes.

Following the reporter assays which identified sequences required for functional promoter activity for each *falcipain *gene, the next task was to identify the nuclear factors binding to these promoter regions. For this purpose, we carried out electrophoretic mobility shift assays (EMSA) using probes ranging between 200 – 400 bps sizes from the 5'upstream regulatory regions of the four *falcipains*. Multiple probes were designed from *falcipain-*2, -2' and -3 promoter regions between -2 kb and -1 kb region, while a single probe was designed from *falcipain-*1 promoter sequence upstream to the transcription start site. Position of each probe for the *falcipains *is shown in fig 5A. Slow migrating protein-DNA complexes were detected for the probes 1.1 (*falcipain-*1), 2.2 & 2.3 (*falcipain-*2), 2'.2 (*falcipain-*2' and 3.2 (*falcipain-*3) on 6% native polyacrylamide gels (fig 5B). Some of these slow moving protein-DNA complexes as indicated by arrows in fig 5B were specific as excess unlabeled probe competed for binding to these complexes. In the case of *falcipain*-2, two sequence elements were able to bind nuclear factor(s). Positions of protein-DNA complexes were apparently different for each probe; thereby suggesting that distinct nuclear factors are involved in regulating the expression of different *falcipain *genes.

### Nuclear proteins interact stage-specifically with falcipains promoters

**Figure 6:**
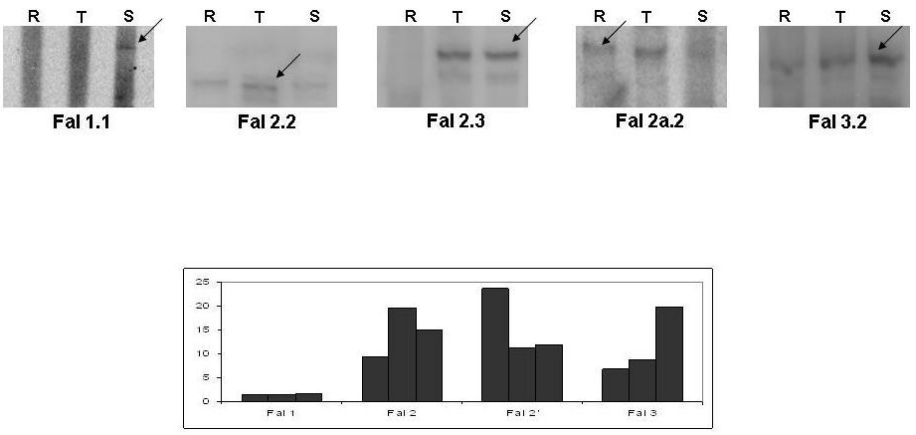
**Stage specific interaction of *falcipain *sequence elements with nuclear extracts.** Nuclear extracts were prepared from synchronous cultures containing ring (R), trophozoites (T) and schizonts (S) stage parasites. Arrows indicate the DNA-protein complexes. Lower panel shows the reporter gene activity of each *falcipain *promoter at three developmental stages (Fig 4, for comparison purposes).

Having established that distinct nuclear factors bind to specific sequences in the promoter regions of the *falcipains*, we further examined the formation of these DNA protein complexes at ring, trophozoite and schizont stages using EMSA analysis. As shown in fig. 6, DNA- protein complexes appeared for each falcipain promoter sequence in stage specific manner. For example, a specific DNA-protein complex was observed for *falcipain-*1 promoter sequence with schizont stage nuclear extract, but not with the ring or trophozoite stage extracts. Likewise, probe 2.3 (*falcipain-2*) was able to bind with nuclear extracts from trophozoite/schizont stages, but not from ring stage. EMSA analysis thus suggested the expression of stage specific nuclear factors which bind to different *falcipain *promoters.

## Discussion

Transcriptional and post-transcriptional regulatory processes have been shown to regulate the expression of parasite proteins and transcriptional regulation appears to be the major regulatory mechanism operating in malaria parasites [5]. It appears that the molecular mechanisms that regulate gene expression in malaria parasite may differ from that of other eukaryotes [1,4]. However, a recent study using bioinformatics approaches has predicted the existence of parasite encoded general transcription factors such as TFIIA, TFIIH, TFIIE α unit, and several subunits of TFIID in *P. falciparum *genome. Another study has identified divergent version of the AP2-integrase DNA binding domain that is present in numerous plant transcription factors in the Apicomplexans [17]. A highly conserved transcription factor of Myb family (PfMyb1) that regulates the expression of a number of parasite proteins has also been described in *Plasmodium falciparum *[12,18].

In malaria parasites, multiple cysteine and aspartic proteases have been reported and they show functional redundancy; these proteases show ability to compensate each other's functions. For example, falcipain-2' takes over the function of falcipain-2 in falcipain-2 knockout *P. falciparum *parasites [16]. The ability to compensate for the individual protein function among these proteases can also be explained by close similarity in their structure and active site residues. These proteases are responsible for hemoglobin degradation in malaria parasite, an important metabolic process central to growth and maturation of parasites [13,19]. However, nothing is known about the transcriptional regulation of these proteases in *P. falciparum*. The current study was performed to identify regulatory regions of four *falcipains *expressed during the asexual blood stages of the parasite.

As *falcipains *promoter sequences have not been characterized previously, we mapped the transcription initiation sites for the four *falcipains *by 5'RACE analysis. The positions of transcription initiation sites differ considerably among four *falcipains*. The start sites were in concordance with the known and predicted transcriptional start sites with all the genes having either A or C as their start sites [20]. A single RNA initiation site was observed for *falcipain-*1, -2 and -3, while two initiation sites were noted for *falcipain*-2'. The significance of two start sites for *falcipain -*2' requires further investigation to determine if alternate transcripts exist. Nevertheless, multiple RNA initiation sites have been reported earlier for *P. knowlesi *circumsporozoite gene, *P. falciparum *MSP-1 gene and *P. yoelii *Py230 gene [21-23]. We did not perform primer extension or RNAse protection experiments to confirm the start sites for the falcipain genes, as the focus of the study was to identify regulatory elements for the falcipain genes. Even though 5'RACE experiment was carried out with good quality of mRNA, there is still a possibility for discrepancies concerning the start sites because of mRNA degradation. We next examined the sequences required for functional promoter activity for each *falcipain *by analyzing the reporter gene activity (luciferase activity) in transient transfection assays. Our data suggests that a 1 kb 5' upstream region from the translational start site is sufficient for the functional promoter activity of *falcipain-*1, while 1.6 to 2 kb upstream sequences of *falcipain-*2,-2'*and *-3 are required to drive significant luciferase expression. We did observe basal level of promoter activities when sequences 1 kb upstream of *falcipain*-2, -2' and -3 were used in the reporter assay. A comparison of transcriptional activity as determined by reporter assays for *falcipain *genes showed that *falcipain-*2' promoter is a strong promoter. Apparently, the *falcipain-*2' promoter represents an intergenic region between *falcipain-*2' to *falcipain-*3. It is possible that this region may also include the 3'UTR sequences for *falcipain*-3.

As falcipains differ in their timing of expression at asexual blood stages of *P. falciparum*, their promoter activities were analyzed at the different blood stages; ring, trophozoite and schizonts. Considerable variations were seen in the *falcipains *promoter activities at the three stages and these variable activities coincided with the expression profiles of these proteins. *Falcipain-2 *promoter showed maximum activity at the trophozoite stage which is in accordance with its expression profile. Similar was the case for *falcipain-*1 and-3. In case of *falcipain-*2', a previous study [16] showed a maximum transcript level for this protein at the schizont stage, while, in the present study, luciferase activity was maximum at the ring stage. Thus, our study suggests that a tight transcriptional regulation governs the stage specific expression of the *falcipains*.

To further elucidate the transcriptional regulation of *falcipains*, we studied the interaction of nuclear proteins with different regions of *falcipain *promoters. Distinct nuclear factors were found to be interacting with specific sequences within each *falcipain *promoters. For example, probes Fal 2.3 and Fal 3.2 formed higher molecular weight complexes than probe Fal 2a.2.

Having established the distinct nature of complexes, the question arose, were these specific DNA/protein complexes stage specific? To answer this, we performed EMSA assays with nuclear extracts prepared from three asexual blood stages of *P. falciparum*. Protein-DNA complexes were formed depending upon the developmental stage of the parasite. In case of *falcipain-*2, two independent sequences (probes 2.2 and 2.3) formed complexes at different stages. Probe 2.2 formed complexes with all the three stage specific nuclear extracts while probe 2.3 formed complexes only with trophozoite and schizont specific nuclear extracts. We hypothesize that probe 2.2 contain sequences that probably bind to general transcription factors/cis-acting elements, while probe 2.3 contain sequences that are part of a specific transcriptional machinery and are crucial for the stage dependent expression of the protein. A closer look at the sequence of probe 2.3 revealed the presence of a TGCAC motif, present exclusively in proteases (see Additional file 1). Probe 2a.2 of *falcipain-*2' formed complexes with nuclear extracts from the ring and trophozoite stage but no complex was observed with the nuclear extract from schizont stage. This is in concordance with the reporter assay that showed maximum activity for *falcipain-*2' promoter at the ring stage. Together these results suggest that the malaria parasite expresses transcription factors and trans/cis regulatory factors in a stage dependent manner that specifically binds to *falcipains *5' upstream sequences. We also analyzed the role(s) of falcipain proteins in regulating the expression of other falcipain proteins (data not shown). However, neither falcipain-1 nor falcipain-2 proteins were able to bind any of the upstream sequence or to a DNA protein complex in an EMSA assay.

Sequence alignment of the nuclear factors binding sequences of *falcipain*s did not reveal a common element among these promoters, nor were homologies seen with many known binding sites of higher eukaryotic transcription factors. However, we did find parasite specific sequence elements in the upstream regions of *falcipains*. Long poly(dA) poly(dT) tracts that have been previously shown to regulate *Pfcam *activity were present in all *falcipains *5'upstream regulatory sequences [24]. The length and density of these tracts varied amongst the four *falcipains*. These poly(dA) poly(dT) tracts have been shown to influence the transcriptional activity in yeast, *Giardia lamblia *and *Dictyostelium discoideum *[25-27]. We also identified a CCAAT box [28] in upstream sequences of *falcipain*-3 gene; however, the significance of this element in *falcipain-*3 needs further investigation. A single G-rich sequence, (A/G)NGGGG(C/A) that is typical of 5'UTR of heat shock genes in *Plasmodium *[28] was present in upstream regions of *falcipain-*2 & -3. In *falcipain 2*, this sequence is present within the EMSA probe (see Additional file 1), while in *falcipain 3*, this motif is present upstream to the EMSA probe. A motif, TGCAC previously identified in a class of functionally related protease was also seen in upstream region of *falcipain-2 *[29]. Thus, sequence analysis and alignment data of the *falcipains *5'upstream regulatory regions suggest that cis-acting factors/transcription factor(s) distinct from that of human host are involved in regulating the expression of *falcipains*. Though, our initial attempts to identify the DNA binding proteins failed, isolation and characterization of parasite's cis-regulatory elements and transcription factors, which are apparently highly divergent from those of other eukaryotes due to the high A+T-richness of *P. falciparum *genome will be valuable to gain insight into the gene regulatory processes in *P. falciparum*.

## Conclusion

In conclusion, we have identified sequences that are essential for functional promoter activity for each of the four *falcipains*. We have also shown that distinct nuclear factors bind to specific sequences of each *falcipain *promoter that in turn may determine the developmental stage specific patterns of *falcipains *expression. Characterization of parasite specific regulatory elements may provide new insights into the biology and pathogenicity of malaria parasite that can reveal new opportunities for intervention.

## Methods

### Parasite culture and transfection

*P. falciparum *3D7 strain was continuously cultured *in vitro *with human erythrocytes (4% hematocrit) in complete RPMI 1640 media (Invitrogen) supplemented with 10% human sera following the protocol described previously [30]. Parasites were synchronized by rounds of Percoll-enrichment of late-stage schizonts, followed by sorbitol treatment using a protocol described by Lambros and co-workers. [31]. Ring stage parasites (parasitemia 5–7%) were transfected as described by Wu and co workers [32]. Transfections were carried out with 75 μg of maxiprep (Qiagen) purified DNA. *Renilla *luciferase vector (pPfrluc) was used as an internal control for all the transfection experiments. Vector pGL2 with the luciferase gene but no promoter (Promega) served as a negative control for all the luciferase assays. Parasites were harvested at different time points, 48 h, 75 h and 90 h post transfection for the analysis of luciferase activity. All the experiments were done in triplicates.

### 5'Rapid Amplification of cDNA Ends Assay (5'RACE)

Total RNA was extracted from 3D7 parasite culture having parasitemia 8–12% using TRIzol reagent. mRNA was extracted from the total RNA using biotinylated oligo dT primers. Following mRNA isolation, the first strand synthesis was done with gene specific primer (GSP1) using SuperScript II Reverse Transcriptase (Invitrogen). TdT tailing was done with column purified cDNA sample and the tailed cDNA was amplified directly by PCR with gene specific primer (GSP2) and abridged anchor primers. In the case of *falcipain 2 *and *2'*, GSP1 and GSP2 were the same as the two genes shared 97% homology. The PCR conditions for the amplification of 5'UTR sequences of different falcipains are listed in Table 1. A second round of amplification using the product obtained from first PCR reaction was done with a primer, GSP3. In the second round of PCR reaction, the initial five cycles had the annealing temperature of 48°C with an initial denaturation of 94°C for 1 min and extension at 68°C for 1 min followed by 30 cycles of PCR conditions given in Table 1. Secondary PCR products were ligated into pGEMT-Easy (Promega) vector, and ligations were transformed into competent JM109 cells. Plasmid DNAs were prepared from individual clones using the Qiagen Miniprep Spin kit and the inserts were sequenced using gene-specific primers.

### Plasmid constructs

All constructs were cloned in pPf86 plasmid (kindly provided by Militello and Dyann F Wirth, Harvard School of Public Health, Boston, MA, USA) having the 5'hsp86 promoter sequences fused to the firefly luciferase open reading frame (ORF). 5' upstream sequences flanking the *falcipain *genes (*Falcipain-1, Falcipain-2, Falcipain-2' and Falcipain-3*) were amplified from genomic DNA with the respective primers using Pfu DNA polymerase (Stratagene) and primers possessing *XhoI *and *NcoI *sites. *XhoI *site was positioned at their terminal end while *NcoI *site was placed in the middle of the reverse primers. This was done for the specific purpose of facilitating PCR as the region immediately upstream to the translational start site was extremely AT rich. The primers were designed from the 5' upstream regions and 6–7 bases from the gene were also included. The *NcoI *site was placed right at translation start site and the bases from the gene was removed at the time of cloning. The list of primers used for PCR amplifications is given in 2. For the 2 kb and 1 kb constructs, the initial five cycles had the annealing temperature of 48°C with an initial denaturation of 94°C for 1 min and extension at 68°C for 1 min followed by 30 cycles of PCR conditions (Table 2). The amplified sequences were cloned into a TA cloning vector, pGEMT easy (Promega) and sequenced. The inserts from pGEMT vector were isolated and cloned upstream of the firefly luciferase reporter gene between the *XhoI *and *NcoI *sites in pPf86 vector. The cloned sequences and junctions were confirmed by restriction mapping and by sequence analysis using automated sequencer (ABI).

**Table 2:** List of primers and PCR conditions used for making luciferase constructs

**Gene**	**Primers**	**Primer sequences 5'-3'**	**PCR conditions**		
			
			**Denaturation**	**Annealing**	**Extension**
**Falcipain 1**	Fal1Luc 1 kb F	CTCGAGATTCATTCTGTGTCATCTGT	94°C 1'	53°C 1.30'	68°C 1.30'
	Fal1Luc 0.5 kb F	CTCGAGGCCTTTTCTTTCTATTTTAAGC	94°C 1'	53°C	68°C 1'
	Fal1Luc R	CCATGTTGTAATCCATGGATTTTTTAT			
**Falcipain 2**	Fal2Luc 2 kb F	CTCGAGTATATTGTATGTAATTTGAGATAATGA	94°C 1'	52°C 1.30'	62°C 2'
	Fal2Luc 1 kb F	CTCGAGTGAATATATAGAAACCTCACTAGG	94°C 1'	55°C 1.30'	68°C 1.30'
	Fal2Luc 0.5 kb F	CTCGAGAATTCATTGCATTTATGTGTTTTCTA	94°C 1'	53°C 1'	68°C 1'
	Fal2Luc R	CATGTGGTAATCCATGGTCTTCAAC			
**Falcipain 2a**	Fal2aLuc 2 kb F	CTCGAGGATGATTTCGCCTTTTATAG	94°C 1'	50°C 2'	65°C 2'
	Fal2aLuc 1 kb F	CTCGAGTATGAATATACATATACACTAGG	94°C 1'	52°C 1.30'	65°C 1.30'
	Fal2aLuc 0.5 kb F	CTCGAGAAAGATAAGCAACATCGATT	94°C 1'	53°C 1'	68°C 1'
	Fal2aLuc R	CATGTGGTAATCCATGGTTAAAA			
**Falcipain 3**	Fal3Luc 2 kb F	CTCGAGCATACAGTTGAAGGGATGATG	94°C 1'	52°C 1.30'	62°C 2'
	Fal3Luc 1 kb F	CTCGAGACAAAGAAGAATATGAACAAAG	94°C 1'	55°C 1.30'	68°C 1.30'
	Fal3Luc 05.kb F	CTCGAGTTCGAAAATTTCCGTATACAT	94°C 1'	53°C 1'	68°C 1'
	Fal3Luc R	CATATGATATTCCATGGTTCAAAC			

### Luciferase assay

To perform the luciferase assay, transfected parasites were harvested by pelleting the infected erythrocytes (300 μl of packed cell volume/culture). The parasite pellets were lysed for 10 min on ice with PBS containing 0.15% saponin. The parasites were centrifuged at 7000 rpm for 10 min at 4°C and the pellets were washed twice with 1% PBS to eliminate residual debris. The pellets were then resuspended in 50 μl of passive lysis buffer (Promega) and kept at RT for 10 min to ensure total lysis and centrifuged at 13,000 rpm. *Renilla *and firefly luciferase activities were measured as described in the Dual Luciferase reporter assay system kit (Promega) using a Berthold luminometer. All experiments were performed at least twice and done in triplicates.

### Preparation of parasite nuclear protein extracts

Nuclear protein extracts were prepared as described by Voss and co-workers [33] with some modifications. About 10 × 10^10 ^parasitized erythrocytes (~10% parasitemia) at the three asexual blood stages, rings, trophozoites or schizonts were lysed in PBS containing 0.15% saponin. The parasites were washed with 1× PBS and resuspended in ice-cold lysis buffer (20 mM HEPES pH 7.8, 10 mM KCl, 1 mM EDTA, 1 mM DTT, 1 mM PMSF, 0.65% NP-40) for 5 min on ice. The lysates were centrifuged at 2500 g for 5 min and the supernatants containing the cytoplasmic proteins were removed. The remaining pellets containing intact nuclei were washed twice with lysis buffer. After washing, the nuclear pellets were resuspended in one pellet volume of extraction buffer (20 mM HEPES pH 7.8, 800 mM KCl, 1 mM EDTA, 1 mM DTT, 1 mM PMSF, 3 uM Pepstatin A, 10 uM leupeptin) and the nuclei were extracted with vigorous shaking at 4°C for 30 min. The extracted nuclei were centrifuged at 13000 g for 30 min. The supernatants containing the nuclear proteins were diluted with one volume of dilution buffer (20 mM HEPES pH 7.8, 1 mM EDTA, 1 mM DTT, 30% glycerol) and stored at -80°C until further use.

### Electromobility Shift Assay (EMSA)

Complementary strands of the probes used for EMSA were labeled with α-^32^P ATP using PCR in a 50 μl reaction mixture. The reaction consisted of 10 ng of template, 10 pmoles of the specific primers (Table 3), 100 μM of each dNTPS, 5 μl of α-^32^P ATP and 1 unit of Taq DNA polymerase (Promega). Labeled probes were purified using QIAquick nucleotide removal kit (Qiagen) and the counts were checked by using scintillation counter.

**Table 3:** List of primers for EMSA probes

**Gene**	**Primer Name**	**Primers 5'-3'**
**Falcipain 1**	Fal 1.1 F	ATTCATTCTGTGTCATCTGT
	Fal 1.1 R	CTATTTATAAAAAAAATTTC
**Falcipain 2**	Fal 2.1 F	TATATTGTATATAATTTGAGATAATG
	Fal 2.1 F	CCCAAGCCGATTATTTTTTTTAAT
	Fal 2.2 F	TAAAAAAAATAATCGGCTTGGG
	Fal 2.2 R	GACCATGCGCATTTACTAG
	Fal 2.3 F	CTAGTAAATGCGCATGGTC
	Fal 2.3 R	TAGTGAGGTTTCTATATATTC
**Falcipain 2a**	Fal 2a.1 F	CCTTAATAAAAAGAAATGAACAA
	Fal 2a.1 R	CACTTTTCCCTATTTTATACTA
	Fal 2a.2 F	TAGTATAAAATAGGGAAAAGTGT
	Fal 2a.2 R	CATATATTATAATTGTTTCTCTTT
**Falcipain 3**	Fal 3.1 F	CATACAGTTGAAGGGATGAT
	Fal 3.1 R	GCATCATAATCAAAATATGGCAT
	Fal 3.2 F	ATGCCATATTTTGATTATGAT
	Fal 3.2 R	GAATGATTAAAATAATAATAAATAAA
	Fal 3.3 F	TTATTATTATTTTAATCATTC
	Fal 3.3 R	CTTTGTTCATATTCTTCTTTGT

EMSA reactions were carried out by incubating 2–5 μg of nuclear proteins with 5 fmol of radiolabeled probe in EMSA buffer (50 mM Tris pH 7.5, 250 mM NaCl, 5 mM MgCl_2_, 2.5 mM EDTA, 2.5 mM DTT, 20% glycerol) containing 500 ng of poly(dI-dC) as nonspecific competitor in a 20 μl reaction volume for 20 min at room temperature. Binding reactions were analyzed on a 6% native polyacrylamide gel in 0.5% TBE. Gels were dried and exposed for autoradiography. Scanning was performed using Typhoon 9210, variable mode Imager from Amersham Biosciences, USA. For competition experiments, the labeled probe was added 10 min after incubation of competitor DNA.

## Authors' contributions

SS carried out all the experimental procedures. PM supervised the work. PM and SS were involved writing the manuscript. VSC was involved in overall supervision and in discussion of the work.

## Supplementary Material

Additional file 1 Sequences of *falcipains *probes used in EMSA analysis. The positions of parasite specific sequence elements known to regulate transcription in *P. falciparum *are highlighted; polydT tracts shaded in green, TGCAC motif shaded in yellow, G-box indicated in purple. The primers used for making probes are underlined. Click here for file

## References

[B1] Callebaut I, Prat K, Meurice E, Mornon Jean-Paul, Tomavo S (2005). Prediction of the general transcription factors associated with RNA polymerase II in *Plasmodium falciparum*: conserved features and differences relative to other eukaryotes. BMC Genomics.

[B2] Bozdech Z, Llinas M, Pullium BL, Wong ED, Zhu J, DeRisi JL (2003). The transcriptome of the intraerythrocytic developmental cycle of *Plasmodium falciparum*. PLOS Biology.

[B3] Walters AP (2003). Parasitology: Guilty until proven otherwise. Science.

[B4] Horrocks P, Dechering K, Lanzer M (1998). Control of gene expression in *Plasmodium falciparum*. Mol Biochem Parasitol.

[B5] Coulson RM, Hall N, Ouzounis CA (2004). Comparative genomics of transcriptional control in the human malaria parasite *Plasmodium falciparum*. Genome Res.

[B6] Hall N, Karras M, Raine DJ, Carlton Kooij TWA, Berriman M, Florens L, Janssen CS, Pain A, Christophides GK, James K, Rutherford K, Harris B, Harris D, Churcher C, Quail MA, Ormond D, Doggett J, Trueman HE, Mendoza J, Bidwell SL, Rajandream MA, Carucci DJ, Yates JR, Kafatos FC, Janse CJ, Barrell B, Turner CM, Waters AP, Sinden RE (2005). A comprehensive survey of the *Plasmodium *life cycle by genomic, transcriptomic, and proteomic analyses. Science.

[B7] Waters AP (1994). The ribosomal RNA genes of *Plasmodium*. Adv Parasitol.

[B8] Scherf A, Hernadez-Rivas R, Buffet P, Bottius E, Bernatar C, Pouvelle B, Gysin J, Lanzer M (1998). Antigenic variation in malaria: In-situ switching, relaxed and mutually exclusive transcription of var genes during inta-erythrocytic development in *Plasmodium falciparum*. EMBO J.

[B9] Deitsch KW, Calderwood MS, Wellems TE (2001). Malaria. Cooperative silencing elements in var genes. Nature.

[B10] Dzikowski R, Frank M, Deitsch K (2006). Mutually exclusive expression of virulence genes by malaria parasites is regulated independently of antigen production. PLoS Pathogens.

[B11] Paton MG, Barker GC, Matsuoka H, Ramesar J, Janse CJ, Waters AP, Sinden RE (1993). Structure and expression of a post-transcriptionally regulated malaria gene encoding a surface protein from the sexual stages of *Plasmodium berghei*. Mol Biochem Parasitol.

[B12] Gissot M, Briquet S, Refour P, Boschet C, Vaquero C (2005). PfMyb1, a *Plasmodium falciparum *transcription factor, is required for intra-erythrocytic growth and controls key genes for cell cycle regulation. J Mol Biol.

[B13] Rosenthal PJ (2004). Cysteine proteases of malaria parasites. Int J Parasitol.

[B14] Greenbaum DC, Baruch A, Grainger M, Bozdech Z, Medzihradszky KF, Engel J, DeRisi J, Holder AA, Bogyo M (2002). A role for the protease falcipain 1 in host cell invasion by the human malaria parasite. Science.

[B15] Kumar A, Kumar K, Korde R, Puri SK, Malhotra P, Chauhan VS (2007). Falcipain-1, a *Plasmodium falciparum *cysteine protease with vaccine potential. Infect Immun.

[B16] Sijwali PS, Rosenthal PJ (2004). Gene disruption confirms a critical role for the cysteine protease falcipain-2 in hemoglobin hydrolysis by *Plasmodium falciparum*. Proc Natl Acad Sci USA.

[B17] Balaji S, Madan Babu M, Iyer LM, Aravind L (2005). Discovery of the principal specific transcription factors of Apicomplexa and their implication for the evolution of the AP2-integrase DNA binding domains. Nuc Acid Res.

[B18] Boschet C, Gissot M, Briquet S, Hamid Z, Claudel-Renard C, Vaquero C (2004). Characterisation of PfMyb1 transcription factor during erythrocytic development of 3D7 and F12 *Plasmodium falciparum *clones. Mol Biochem.

[B19] Coombs GH, Goldberg DE, Klemba M, Berry C, Kay J, Mottram JC (2001). Aspartic proteases of *Plasmodium falciparum *and parasitic protozoa as drug targets. Trends Parasitol.

[B20] Watanabe J, Wakaguri H, Sasaki M, Suzuki Y, Sugano S (2007). Comparasite: a database for comparative study of transcriptomes of parasites defined by full-length cDNAs. Nuc Acid Res.

[B21] Altaba RA, Ozaki LS, Gwadz RW, Godson NG (1987). Organization and expression of the *Plasmodium knowlesi *circumsporozoite antigen gene. Mol Biochem Parasitol.

[B22] Lanzer M, Bruin D, Ravetch JV (1992). A sequence element associated with the *Plasmodium falciparum *KAHRP gene is the site of developmentally regulated protein-DNA interactions. Nuc Acid Res.

[B23] Lewis AP (1990). Sequence analysis upstream of the gene encoding the precursor to the major merozoite surface antigens of *Plasmodium*. Mol Biochem Parasitol.

[B24] Polson HEJ, Blackman MJ (2005). A role for poly(dApoly(dT) tracts in directing activity of the *Plasmodium falciparum *calmodulin gene promoter. Mol Biochem Parasitol.

[B25] Sun CH, Tai JH (1999). Identification and characterization of a ran gene promoter in the protozoan pathogen *Giardia lamblia*. J Biol Chem.

[B26] Ong SJ, Huang LC, Liu HW, Chang SC, Yang YC (2002). Characterization of a bidirectional promoter for divergent transcription of a PHD-zinc finger protein gene and a ran gene in the protozoan pathogen *Giardia lamblia*. Mol Micro.

[B27] Kimmel AR, Firtel RA (1994). Sequence organization in *Dictyostelium*: unique structure at the 5'-ends of protein coding genes. Nucleic Acid Res.

[B28] Militello KT, Dodge M, Bethke L, Wirth DF (2004). Identification of regulatory elements in the *Plasmodium falciparum *genome. Mol Biochem Parasitol.

[B29] Gunasekera AM, Myrick A, Militello KT, Sims JS, Dong CK, Gierahn T, Le Roch K, Winzeler E, Wirth DF (2007). Regulatory motifs uncovered among gene expression clusters in *Plasmodium falciparum*. Mol Biochem Parasitol.

[B30] Trager W, Jenson JB (1976). Human malaria parasites in continuous culture. Science.

[B31] Lambros C, Vanderberg JP (1979). Synchronization of *Plasmodium falciparum *erythrocytic stages in culture. J Parasitol.

[B32] Wu Y, Sifri CD, Lei H-H, Su X, Wellems TE (1995). Transfection of *Plasmodium falciparum *within human red blood cells. Proc Natl Acad Sci USA.

[B33] Voss TS, Mini T, Jenoe P, Beck HP (2002). *Plasmodium falciparum *possesses a cell cycle-regulated short type replication protein A large subunit encoded by an unusual transcript. J Biol Chem.

