# Supplementary material for: Distinct and stage specific nuclear factors regulate the expression of falcipains, Plasmodium falciparum cysteine proteases
Source: BMC Mol Biol. 2008 May 14;9:47. doi: 10.1186/1471-2199-9-47 (PMC2409366; doi:10.1186/1471-2199-9-47)
Supplement: Additional file 1 — Sequences of falcipains probes used in EMSA analysis. The positions of parasite specific sequence elements known to regulate transcription in P. falciparum are highlighted; polydT tracts shaded in green, TGCAC motif shaded in yellow, G-box indicated in purple. The primers used for making probes are underlined. [file 1471-2199-9-47-S1.doc]

**Fal1.1**

ATTCATTCTGTGTCATCTGTTTTTTTTTTTTCTTTATTTTTATTTTCTTTATATATATACATTTATTGTAATATATTTATAAATTAATATTATATTTAAAATGGTATAATAAAATATAAATTAAAAATTTAATATGGATGATTTTTAAAAAGATATATATATATATATATATATATATATTATTTGGATCGCTATATATTTTATTTTATATATATTATAATATACATATAAAAAATAATATATAATTTTATATATATATTAAAAATATGTATATAATATAAAAATATATATTTATTATATCTCTATACATAATATAATATATTAAAAAAAGAAAAAAAAAATTATATAAAAAAATATACATTTTTGCTATATATTATTTTTATATATAAATATTTTTATTTTATAATGAAATTTTTTTTATAAATAG

**Fal2.1**

ATTAAAAAAAATAATCGGCTTGGGTTTTTTTTTTGCTTTTTTTTTTTTTTTTTCTTTTTTCTCTATTTCGTAAGTATATTATATAACAACTGGTCGGGCTTTATATTATTATATATTAATTTATAAGGGAAAATAAAAAACAAAAAATTAAAAATATAAAAGAACAAACAATGTTAATAATTATTTATGATAATTAAAGGGGAAAAAAAAATATATATAAAATATTATATTATATAATAATATATATAATATTATATATATATATATTTATATCTAGTAAATGCGCATGGTC

**Fal2.2**

CTAGTAAATGCGCATGGTCCTTAAAATATATCGTTATATATTATATATATATATATATATATATATATATATATTATATATTATTATAATATTTATATTTTTTATTTTCTTAATATGGGTAAGTTTTATTTTTATAGTGAATATTTTTTTTATATATTTATTTTGAACTTTTCACTAAAGAACTAAAATATATGTGATACGTACATAAATATTATGCACTATAAAAAAAGAAATGACTCAAAAAATATATGAATATATAGAAACCTCACTA

**Fal2a.2**

AGTATAAAATAGGGAAAAGTGTCTTCGTATTCAATTTTTTAAATATTTAAAAATAATATAATACTAAATATATGTTATTTTTCATGTGCTATTATTTTTTTTTTTTTTTTTATATTTTTTTTTATTTATATTCTTTTTTTTACAAGTCAGTCATTCTATTTATTTATTTATGTATTTTTTTTTTTTTTTTTTTTATCTAAATTTTTCTATAAAAAAAAGAAAAAATATTTGGCATATATATATTATATACAAAGAGAAACAATTATAATATATG

**Fal3**

ATGCCATATTTTGATTATGATGCTTTTTTTTTTTTTTTTTTTTTTTAGTTAACTTTGATTTATATAATTATTTATAATATATAAAAAAAAAAATATTATTTAAAATAAAAAAATATATATTTTAAGTAGCTTACATAAATAAAGATAAATTTTATAAATACTTCAATTTAAAAAAAATATGGACCACGAATAATGGATAGTTTTGACATAATTTTTTTCATATATAATATAATATGGACTTTTATTATTAAATACAAATAAACATATAATAAAGAATATTAATTTATTTATTATTATTTTAATCATTC

Fig S1
